# Transition in plant–plant facilitation in response to soil water and phosphorus availability in a legume-cereal intercropping system

**DOI:** 10.1186/s12870-022-03706-6

**Published:** 2022-06-28

**Authors:** Shuang-Guo Zhu, Zheng-Guo Cheng, Hai-Hong Yin, Rui Zhou, Yu-Miao Yang, Jing Wang, Hao Zhu, Wei Wang, Bao-Zhong Wang, Wen-Bo Li, Hong-Yan Tao, You-Cai Xiong

**Affiliations:** 1 State Key Laboratory of Grassland Agro-Ecosystems, College of Ecology, Lanzhou University, Lanzhou, 730000 China; 2 School of Ecology and Environmental Science, Yunnan University, Kunming, 650091 China

**Keywords:** Biodiversity, Competition, Coexistence, Rhizosphere, Phosphatase

## Abstract

**Background:**

The tradeoff between negative and positive interactions of facilitated species and facilitators may depend on the degree of resource availability in agroecosystems. However, the rhizospheric mechanisms driving trade-offs that occur along phosphorus (P) and water availability gradients have not yet been systematically clarified. We established three types of root isolation conditions (no barrier, nylon barrier and solid barrier) at different P and water addition levels to address the above issue in a maize-grass pea intercropping system.

**Results:**

The total yield and biomass net effect (NE) and the relative interaction index (RII) were significantly higher than 0 under all environmental conditions, demonstrating that plant-plant interactions generated positive effects in the intercropping system. The maize yield and biomass RII were 0.029–0.095 and 0.018–0.066, respectively, which indicated that maize growth was constantly facilitated. However, the RII for grass pea yield and biomass exhibited a different trend in comparison with maize. It was higher than 0 (as the facilitated species) under low soil P and moisture conditions and transitioned to values lower than 0 (facilitator species) under high P and moisture conditions, which showed that the type and intensity of plant-plant interactions steadily shifted with the applied stressors. Direct interactions decreased the maize rhizospheric soil pH by 1.5% and 1.9% under Low-P conditions. Notably, the rhizospheric soil acid and alkaline phosphatase secretions of maize and grass pea increased by 17.4–27.4% and 15.3–27.7%, respectively, in P-deficient soils. These results show that plant-plant interactions can effectively relieve P stress by mineralizing organophosphorus in P-deficient soils. Furthermore, the above tendency became more pronounced under drought-stressed conditions. The nylon barrier partially restricted the exchange and utilization of available nutrients and decreased the total yield and biomass by 1.8–7.8% and 1.1–7.8%, respectively. The presence of a solid barrier completely restricted interspecific rhizospheric interactions and decreased the total yield and biomass by 2.1–13.8% and 1.6–15.7%, respectively. Phytate and KH_2_PO_4_ addition intensified asymmetric interspecific competition, and grass pea was consistently subjected to competitive pressures.

**Conclusion:**

Briefly, the tradeoff between facilitation and competition was driven by rhizospheric interactions, and the transition in the intensity and type of interaction was highly dependent on resource availability in a biologically diverse system.

**Supplementary Information:**

The online version contains supplementary material available at 10.1186/s12870-022-03706-6.

## Introduction

Species diversity is crucial for primary production in natural and agricultural ecosystems [1, 2]. In comparison with intensive monocultures, biologically diverse communities generally display productivity advantages, which are frequently attributed to facilitative plant–plant interactions and resource acquisition benefits [3, 4]. Substantial amounts of biodiversity data are available on grassland and forest ecosystems on this topic, whereas the relationship between species diversity and productivity along resource gradients in agroecosystems has not yet been systematically investigated [5]. A widely accepted concept is that competition negatively impacts plant growth due to allelopathic effects and due to limited resource availability [6], while facilitation promotes adjacent species growth by improving environmental conditions [7].

Intercropping involves the cultivation of a planned combination of multiple crop species in the same field, and it has been proven to increase yield and resource acquisition, as well as lower production risks [8, 9]. Maize is a major food crop, and grass pea shows strong drought tolerance in the rain-fed agricultural areas of China’s Loess Plateau, consequently, the maize-grass pea intercropping system was selected as the subject of this study. In biologically diverse systems, competitive and facilitative interactions occur simultaneously [10]. Generally, species with a strong abilities to remediate stressful conditions (P and water deficiency) can act as facilitators in agroecosystem [4, 11], and they increases the frequency of positive effects and accordingly offsets negative effects [12].

Extensive research has shown that in diverse agroecosystems, root interactions appear to be more vital than aboveground interactions for determining crop productivity [13, 14]. Interspecific rhizospheric interactions are likely to influence plant physiology, species composition, community structure and microorganism activity [15, 16]. Consequently, interspecific belowground interactions are crucial to achieving better community establishment and ecosystem services [17]. It is feasible and efficient to explore the nature and strength of interspecific root interactions by using pot assays with nylon and solid barriers [18]. These root isolation methods can provide key evidence for the trade–offs between facilitated species and facilitators in agroecosystems [19].

Plants have developed different survival strategies to cope with stressful conditions, and these strategies vary in type and degree among different species [20]. Positive and negative effects can be determined by the environment created by one species and its fitness to another species [21]. The stress gradient hypothesis (SGH) illustrates that plant–plant interactions are context dependent, with competitive interactions dominating in favourable environments and facilitative interactions dominating in stressful environments [22]. In intercropping systems, this hypothesis would predict the dominance of competitive interactions under high nutrient inputs to soils, and predict a potentially greater importance of facilitative interactions under low input conditions [23, 24]. The net effect (NE), relative interaction index (RII) and relative competition intensity (RCI) are three key parameters that allow the assessment biodiversity advantages. NE represents the yield or biomass produced in excess of what is expected, RII evaluates the strength of plant–plant interactions in intercropping systems based on monoculture and species proportion per unit area, and RCI indicates the interspecific and intraspecific competitive ability of a species [3, 25].

Previous studies on this topic have mainly focused on a general facilitation in P-deficient soils without considering soil water and P gradients in the cereal–legume intercropping systems [26, 27]. It is necessary to uncover the driving mechanisms for symmetric and asymmetric facilitation and the functional similarities in mixed species ecosystems. Thus, we hypothesized that nylon and solid barriers would affect plant–plant interaction patterns and productivity outcomes. The major objectives of this study were as follows: 1) to determine the plant growth and rhizospheric soil properties of two crop species in an intercropping system; 2) to identify how plant–plant interaction intensity and type shift with soil water and P gradients; and 3) to reveal the driving mechanisms behind rhizospheric interactions that determine the trade–offs in plant facilitation. The results of this study enhances our understanding of how rhizospheric interactions and the physical environment drive trade–offs in plant facilitation within plant communities.

## Result

### Net effect (NE), relative interaction index (RII) and relative competition intensity (RCI) in response to P and water gradients

**Fig. 1:**
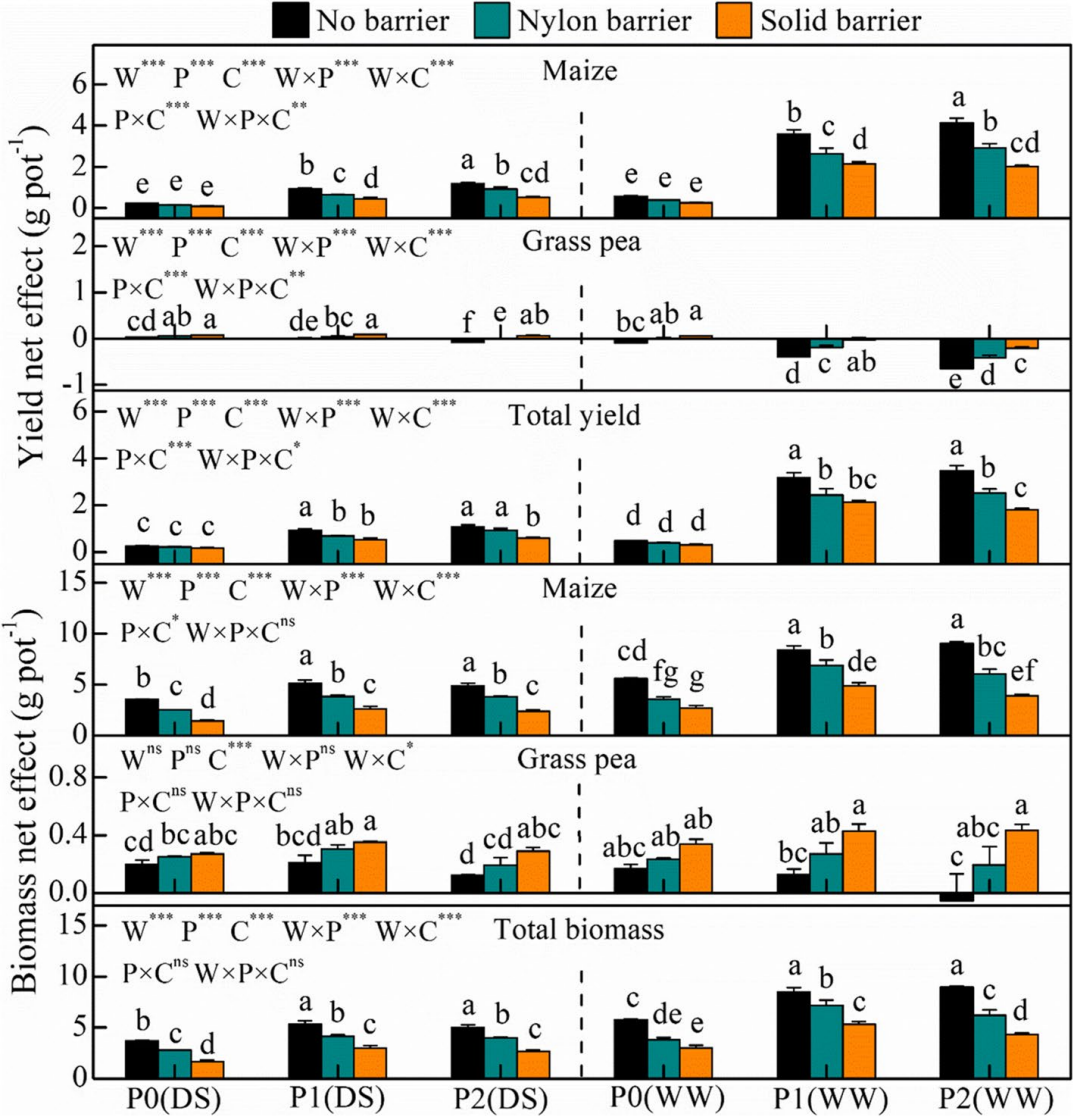
Yield and biomass net effect of maize and grass pea in response to three P treatments (P0-P2) and two water treatments (drought stress (DS) and well-watered (WW)) under three cropping patterns (no barrier, nylon barrier and solid barrier) in maize-grass pea intercropping system. *n* = 5, bars with different lowercase letters (a, b) indicate significant difference (*P* < 0.05). W: water treatment, P: phosphorus treatment, C: cropping pattern. ***, **, *, ns, significant difference at 1‰, 1%, 5% and not significant at 5% level, (the same in the below)

In this study, rhizospheric soil water and P availability showed a significant effect on the total yield and biomass NE (Fig. 1). In comparison with drought–stressed conditions, total yield and biomass NE increased by 208% and 64.4%, respectively, under well-watered conditions. P1 and P2 increased the total yield NE by 439% and 465% and increased the total biomass NE by 60.7% and 50.1% compared with P0, respectively. The results showed that the NE of maize was significantly higher than 0, with the yield NE ranging from 0.1–4.13 g pot^−1^ and biomass NE reaching 1.4–9.0 g pot^−1^. However, grass pea yield and biomass NE showed different trends in comparison with maize. The grass pea yield NE was lower than 0 for P1-P2 under well-watered conditions and was higher than 0 under other conditions.

Root isolation treatments substantially affected the NE of total yield and total biomass, and the highest indices were observed when there were no barriers between the maize and grass pea root systems. The nylon barrier decreased the total yield and biomass NE by 14.4–27.85% and 15.6–34.0%, respectively. The solid barrier decreased the total yield and biomass NE by 39.9–56.5% and 37.6–54.5%, respectively. In particular, root isolation treatments had different impacts on the NE of the two species in the intercropping system. Maize yield and biomass NE were highest when there was no barrier; the nylon barrier decreased the yield and biomass NE by 19.3–32.3% and 17.6–36.3%, respectively, and the solid barrier decreased the yield and biomass NE by 39.9–56.5% and 41.8–59.6%, respectively. Conversely, the nylon and solid barriers significantly increased the grass pea yield and biomass NE indices and decreased the negative effect of plant–plant competition on grass pea.

**Fig. 2:**
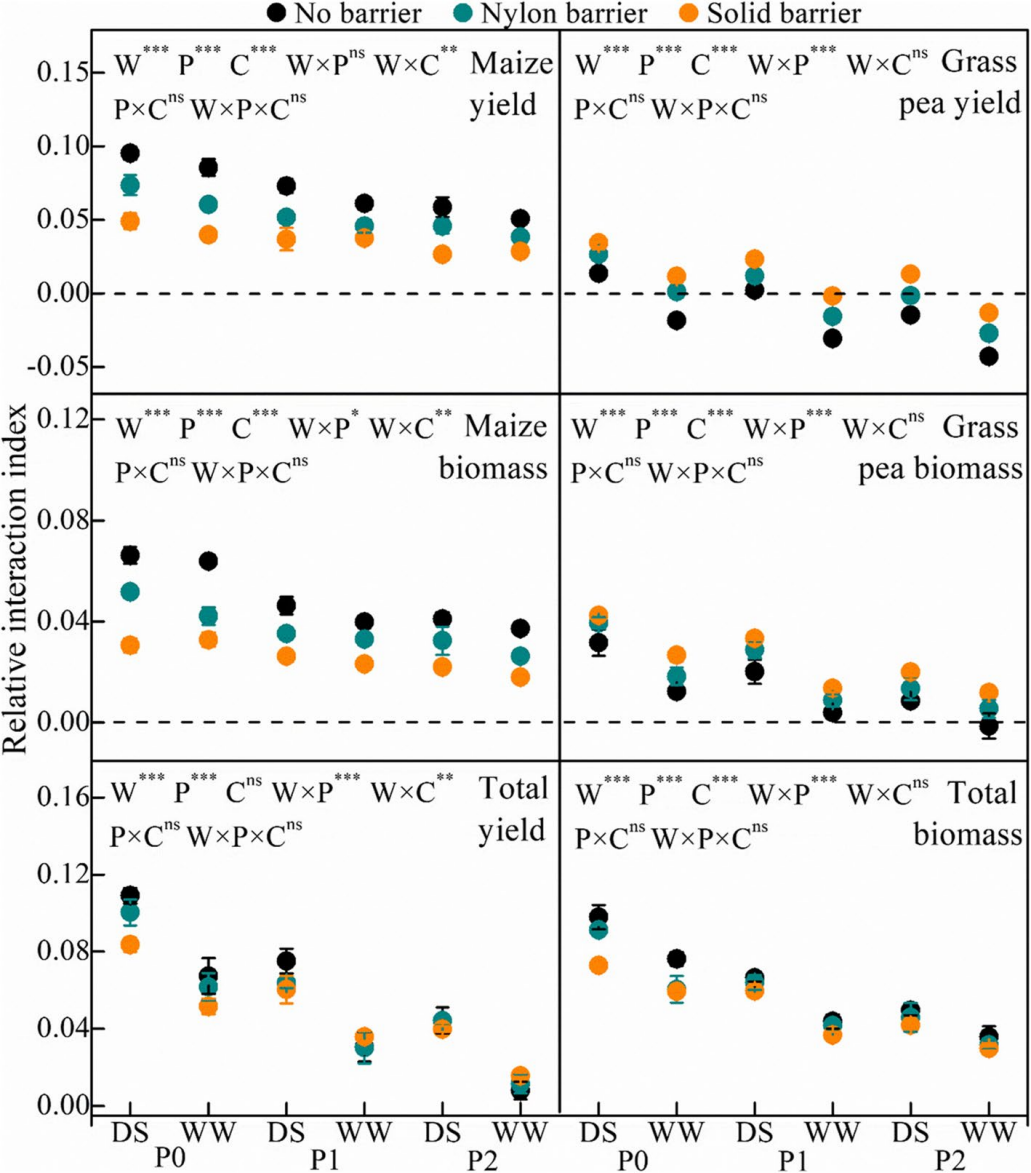
Yield and biomass relative interaction index of maize and grass pea in response to three P treatments and two water treatments under three cropping patterns in maize-grass pea intercropping system

RII exhibited an opposite trend to the NE in that the index decreased with increasing P and water availability (Fig. 2). For total yield and biomass, the indices were greater than 0 under all growth conditions (0.008–0.155 for total yield and 0.030–0.098 for total biomass). Maize grain yield and biomass RII were significantly higher than 0 (0.029–0.095 for yield and 0.018–0.066 for biomass); the highest value was observed under P0, and P1-P2 significantly decreased the RII. Notably, well-watered soils decreased the RII in comparison with drought–stressed soils under the same P condition. Under P0 conditions, the nylon barrier decreased maize yield and biomass RII by 22.6–29.4% and 21.9–34.0%, respectively, and the solid barrier reduced the yield and biomass RII by 48.4–53.6% and 48.7–53.9%, respectively. Under P1 conditions, the nylon barrier reduced the yield and biomass RII by 25.2–29.1% and 17.1–24.0%, respectively, and the solid barrier decreased the yield and biomass RII by 38.6–49.5% and 41.7–43.4%, respectively. Under the P2 condition, the nylon barrier decreased the yield and biomass RII by 22.2–24.3% and 21.0–29.8%, respectively, and the solid barrier decreased the yield and biomass RII by 43.6–54.7% and 46.3–51.7%, respectively.

The root isolation treatments exhibited opposite effects on the two species, and the yield and biomass RII of grass pea were the lowest under the no–barrier conditions. The nylon barrier and solid barrier significantly increased the grass pea RII, and the highest RII value was observed under the solid barrier treatment. The grass pea yield RII was significantly higher than 0 for P0 under drought stress and was lower than 0 in the high P and water treatments under no root barrier applied in the intercropping system. The nylon barrier slightly increased the grass pea yield RII, and the solid barrier significantly increased the index. All grass pea biomass RII values were significantly higher than 0 except under P2 in the well-watered treatment.

**Fig. 3:**
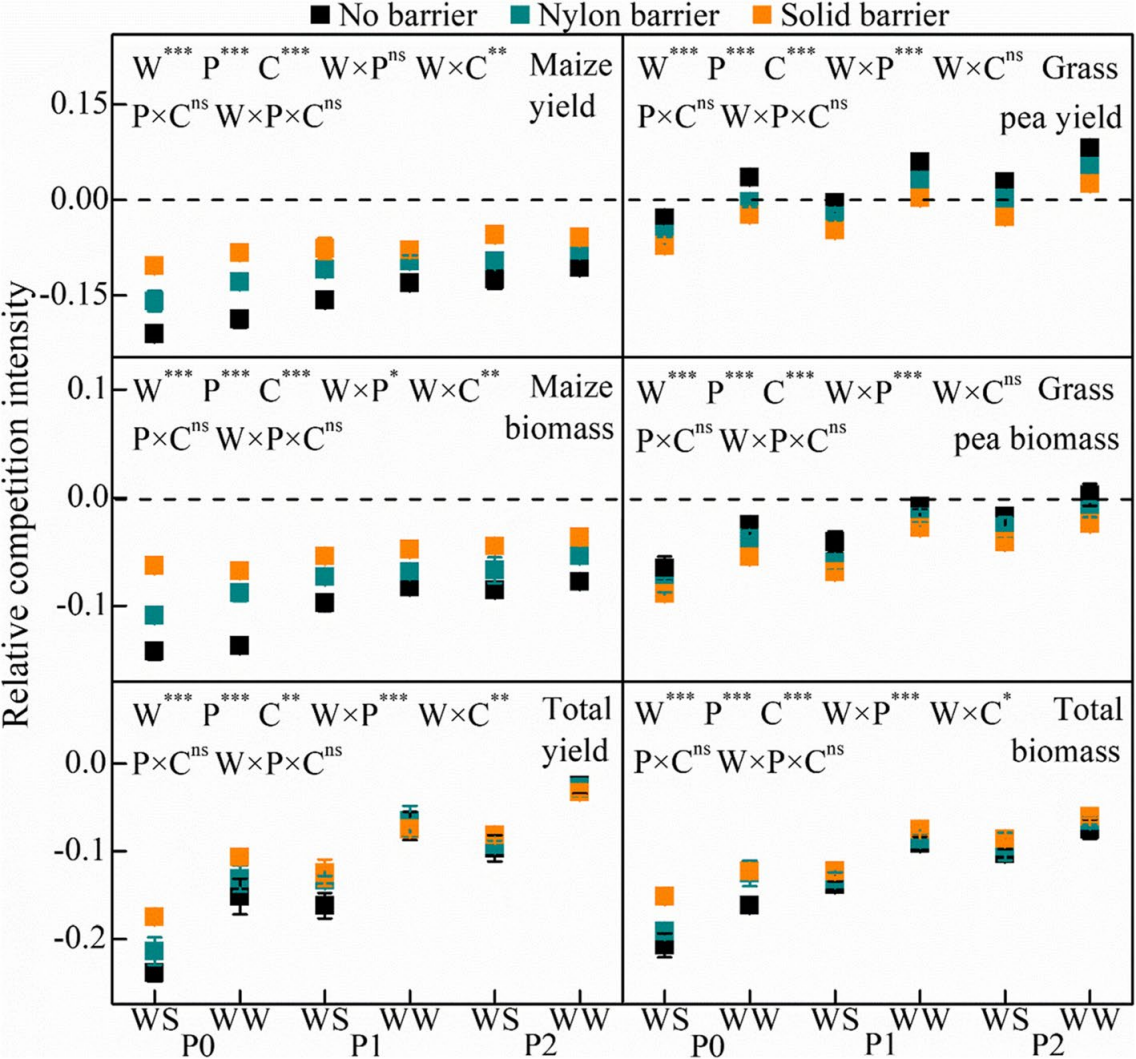
Yield and biomass relative competition intensity of maize and grass pea in response to three P treatments and two water treatments under three cropping patterns in maize-grass pea intercropping system

The RCI under drought stress was lower than that under well-watered conditions (Fig. 3), and P0 had the lowest RCI under the same water conditions. The yield and biomass RCI of maize were significantly lower than 0, and the RCI of grass pea yield was significantly higher than that of maize under the same treatments. The RCI of grass pea biomass was significantly lower than 0 except under P2 in the well-watered treatment. Maize yield and biomass RCI were lowest under the no–barrier condition. The nylon barrier increased the RCI and the highest value was observed in the solid barrier condition. The RCI of grass pea yield and biomass was different from that of maize; the highest RCI was obtained in the no–barrier treatment, followed by the nylon barrier treatment, and the lowest RCI was observed in the solid barrier treatment.

### Rhizosphere soil pH, phosphatase, STP and Olsen-P in response to plant–plant interactions under different environmental conditions

**Fig. 4:**
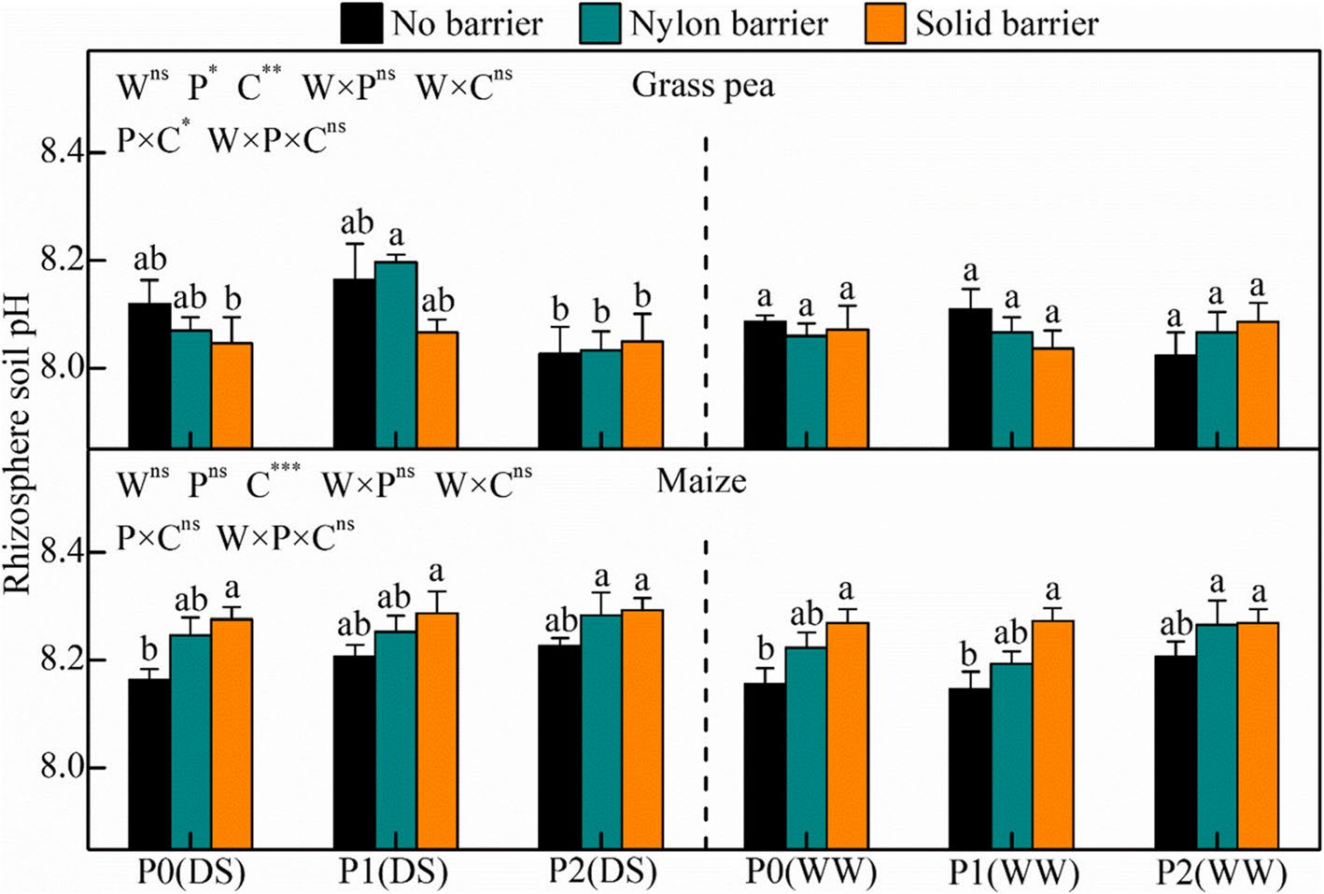
Rhizosphere soil pH of maize and grass pea in response to three P treatments and two water treatments under three cropping patterns in maize-grass pea intercropping system

**Fig. 5:**
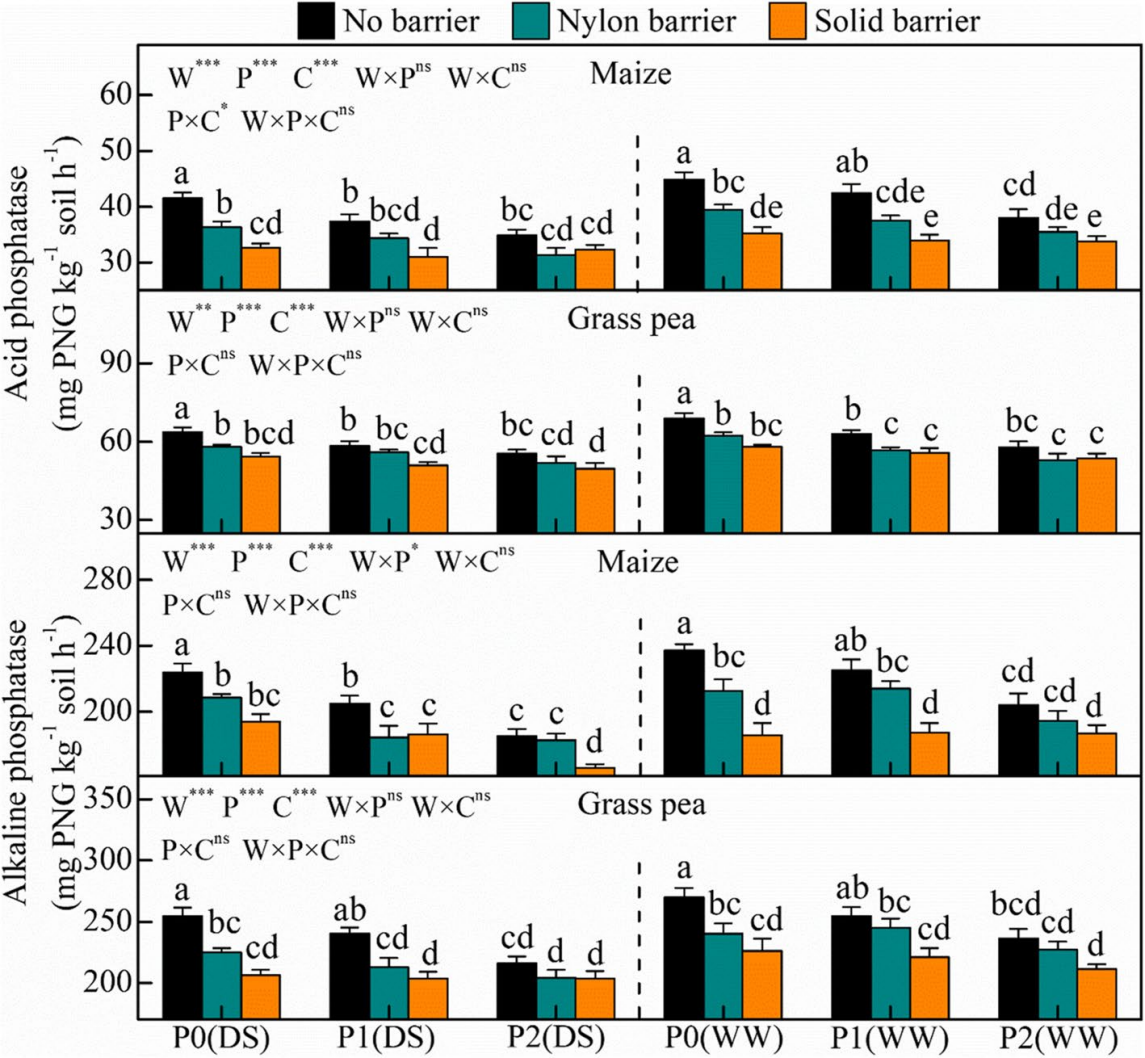
Rhizosphere soil acid and alkaline phosphatase of maize and grass pea in response to three P treatments and two water treatments under three cropping patterns in maize-grass pea intercropping system

In this study, the pH of the grass pea rhizospheric soil was lower than that of maize (Fig. 4). Root interactions significantly decreased the maize rhizospheric soil pH for P0 under drought stress and well-watered conditions and for P1 under well-watered conditions. The secretions of acid and alkaline phosphatase in the rhizosphere of grass pea were significantly stronger than those in maize, and the activities of alkaline phosphatase were relatively greater than those of acid phosphatase (Fig. 5). The amount of secreted acid phosphatase was 51.9–67.9 mg PNG kg^−1^ soil h^−1^in the rhizospheric soil of grass pea and 31.0–48.3 mg PNG kg^−1^ soil h^−1^ in that of maize. Similarly, the alkaline phosphatase secretion reached 204–270 mg PNG kg^−1^ soil h^−1^ in the rhizospheric soil of grass pea and 166–237 mg PNG kg^−1^ soil h^−1^ in that of maize. Direct and indirect root interactions verified the critical role of interspecific plant facilitation on rhizosphere phosphatase secretions, especially under P-deficient conditions. Under P0, the nylon barrier decreased intercropped maize and grass pea rhizospheric acid phosphatase by 7.2–14.3% and 4.3–11.1%, respectively, and reduced alkaline phosphatase by 4.8–11.5% and 3.9–13.1%, respectively. The solid barrier decreased intercropped maize and grass pea rhizospheric acid phosphatase by 7.9–27.3% and 8.0–18.9%, respectively, and reduced alkaline phosphatase by 9.1–27.7% and 6.0–23.4%, respectively.

**Fig. 6:**
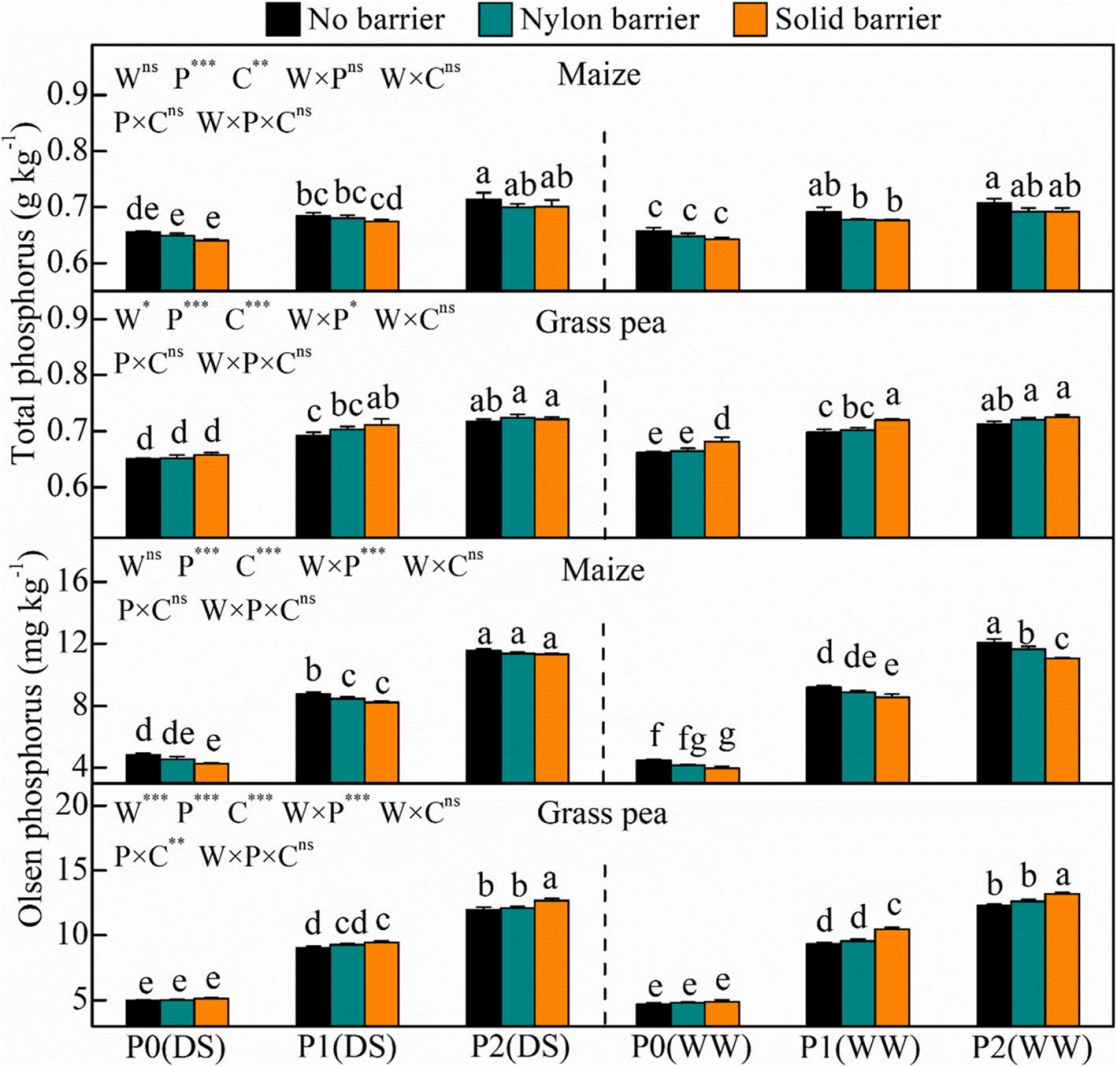
Rhizosphere soil total phosphorus and Olsen phosphorus of maize and grass pea in response to three P treatments and two water treatments under three cropping patterns in maize-grass pea intercropping system

The STP and Olsen-P values in the rhizospheric soil of both species were lowest under P0 (Fig. 6). KH_2_PO_4_ application resulted in higher STP and Olsen-P values under the same water treatment. The solid barrier significantly increased the grass pea rhizospheric soil STP in P1 under drought stress and in P0-P1 under well-watered conditions. Furthermore, the solid barrier decreased the maize Olsen-P value under P0 and P1 by 11.4% and 6.2%, respectively, but significantly increased the grass pea Olsen-P value under P1 and P2 by 5.0% and 5.9%, respectively, under drought–stressed conditions. The solid barrier significantly decreased maize Olsen-P in all P treatments by 6.9–11.5% and increased grass pea Olsen-P in P1 and P2 by 12.0% and 7.6%, respectively, under well-watered conditions.

### Plant yield, biomass, nitrogen uptake, phosphorus uptake, biomass allocation and water use efficiency (WUE)

**Fig. 7:**
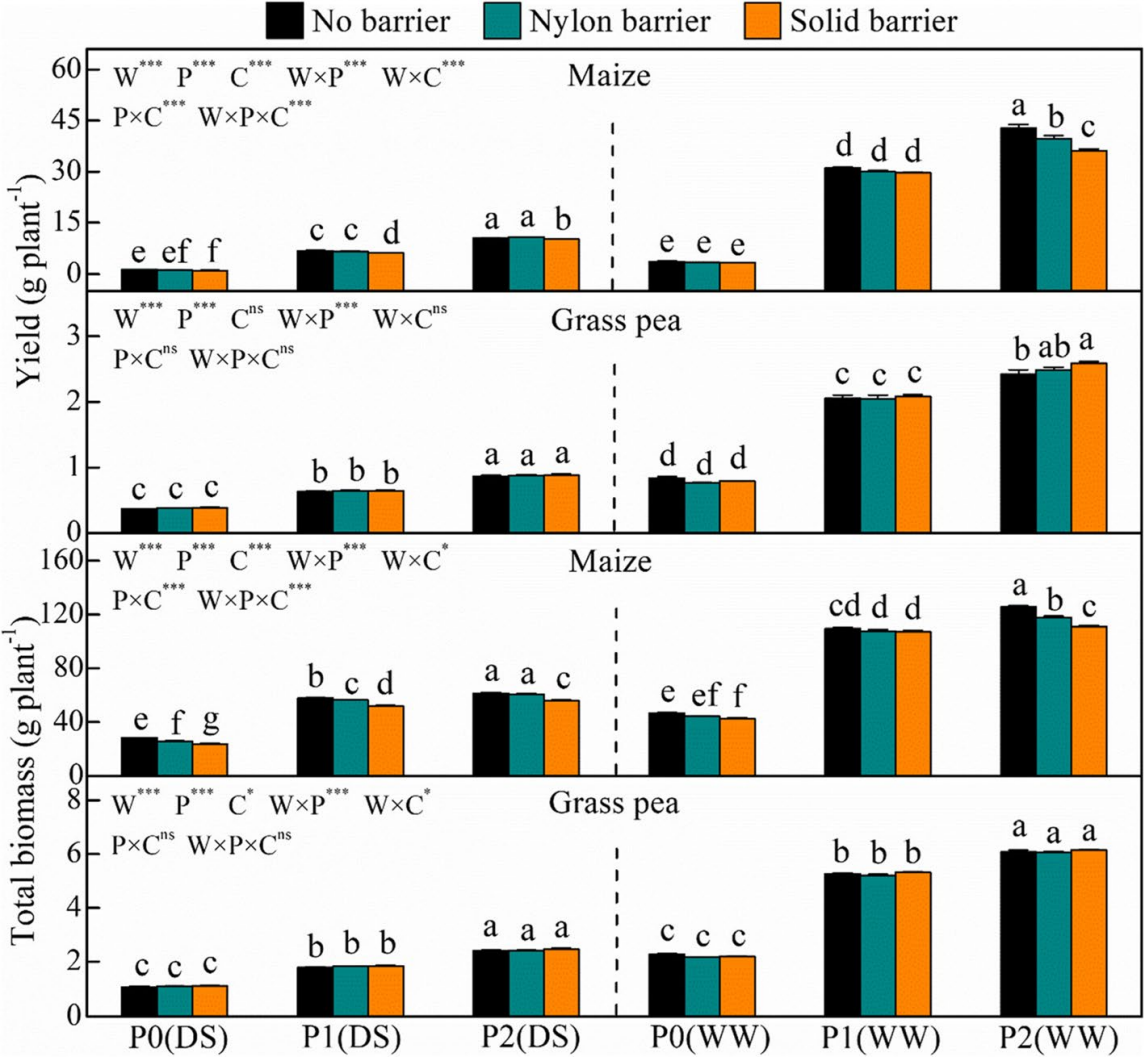
Yield and total biomass of maize and grass pea in response to three P treatments and two water treatments under three cropping patterns in maize-grass pea intercropping system

The mixed species cultivation increased the total yield and biomass by 5.3–11.9% and 6.1–10.4%, 3.7–8.1% and 3.8–6.9%, and 1.7–4.8% and 3.0–5.1% under the P0, P1 and P2 conditions, respectively (Fig. 7). Direct and indirect root interactions exerted opposite effects on the productivity of the two species. Compared with the no–root–barrier treatments, the nylon barrier and solid barrier treatments decreased maize yield by 7.6% and 15.3%, respectively, for P2 under the well-watered treatment, and the solid barrier treatment decreased maize yield by 19.4% in P0, 8.6% in P1 and 3.1% in P2 under drought–stressed conditions. In contrast, the solid barrier significantly increased grass pea yield by 6.7% in P2 under well-watered conditions. The solid barrier reduced maize biomass by 15.6%, 10.4%, and 9.5% in P0-P2, respectively, under drought–stressed conditions, while biomass decreased by 9.1% and 11.6% under P0 and P2, respectively, under well-watered conditions. The nylon barrier decreased maize biomass by 8.5% and 2.6% in P0 and P1 under drought stress, respectively, and decreased by 6.3% for P2 under the well-watered treatment.

**Fig. 8:**
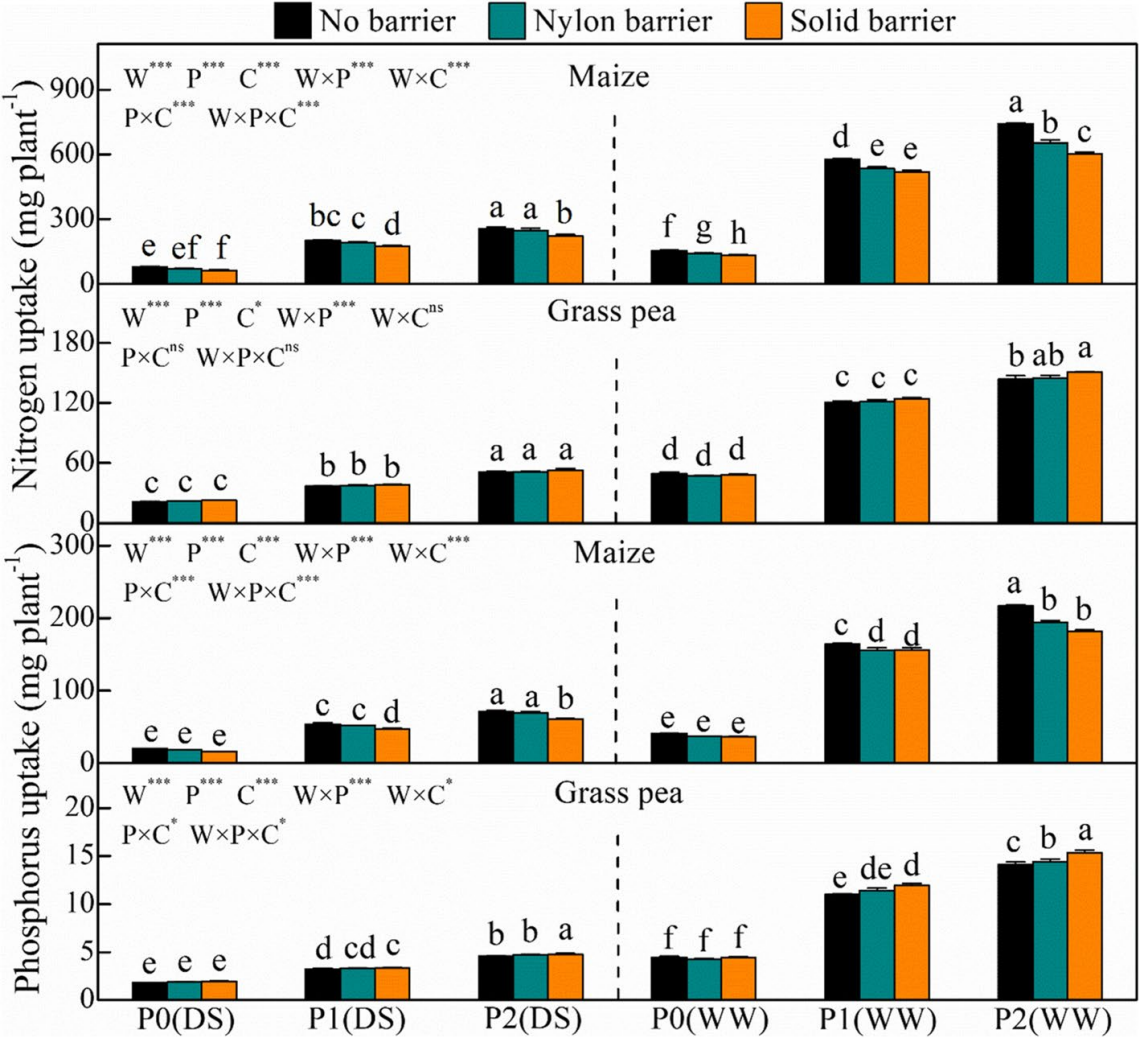
Nitrogen and phosphorus uptake of maize and grass pea in response to three P treatments and two water treatments under three cropping patterns in maize-grass pea intercropping system

Alternatively, P and water addition significantly increased the nitrogen (N) and P uptake of the two species (Fig. 8). In comparison with P0, P1 increased maize N and P uptake by 169–283% and 179–313%, and P2 increased maize N and P uptake by 243–367% and 266–413%, respectively. Similarly, P1 increased grass pea N and P uptake by 72.2–152% and 77.6–161%, respectively, and P2 increased it by 139–203% and 151–233%, respectively. The nylon and the solid barriers showed opposite effects on nutrient accumulation in the two species. The nylon barrier decreased maize N and P uptake by 3.5–12.5% and 2.8–10.8%, respectively, and the solid barrier decreased maize N and P uptake by 10.1–21.2% and 4.7–19.1%, respectively. In contrast, the nylon and solid barriers increased the N and P accumulation of intercropped grass pea.

On the other hand, the maize biomass allocation pattern was more significantly affected by rhizospheric nutrient availability than that of grass pea (Fig. S1). Under well-watered and sufficient–P conditions, maize had greater reproductive organ formation. Under drought conditions, maize grain yield increased by 161% for P1 and increased by 288% under P2. P1 and P2 increased the grain biomass allocation by 258% and 328%, respectively, under well-watered conditions. However, root isolation methods had little effect on the biomass allocation of both species.

The soil water, P and root barriers substantially influenced the yield and biomass WUE of both species (Table 1). WUE significantly increased with increasing soil water and P application. Under drought stress, P1 increased maize yield and biomass WUE by 290% and 49%, respectively, and those of grass pea increased by 19.5% and 16.4%, respectively. P2 increased maize yield and biomass WUE by 395% and 28%, respectively, and grass pea increased yield and biomass WUE by 30.6% and 24.0%, respectively. Under well-watered conditions, P1 increased the yield and biomass WUE of maize by 538% and 78%, those of grass pea increased by 53.5% and 40.8%; and P2 increased maize WUE by 715% and 90.2%, the grass pea WUE increased by 88.2% and 64.8%, respectively. The nylon barrier reduced maize yield and biomass WUE by 2.0–12.8% and 1.9–9.1%, respectively. Maize yield and biomass WUE were reduced by 1.5–17.8% and 6.9–13.9%, respectively, with the solid barrier. In contrast, the nylon barrier increased grass pea yield and biomass WUE by 0.4–5.8% and 1.0–10.0%, respectively, and the solid barrier increased the grass pea yield and biomass WUE by 3.8–9.4% and 3.3–12.0%, respectively.

**Table 1:** Yield and total biomass water use efficiency (WUE) of maize and grass pea in response to three P treatments (P0–P2) and two water treatments (drought stress (DS) and well-watered (WW)) under three cropping patterns (no barrier, nylon barrier and solid barrier) in maize-grass pea intercropping system

Treatments		Maize yield (WUE)	Maize biomass (WUE)	Grass pea yield (WUE)	Grass pea biomass (WUE)
P0 (DS)	No barrier	0.105e	2.242e	0.145e	0.426f
	Nylon barrier	0.092f	2.038f	0.148de	0.436ef
	Solid barrier	0.086f	1.930f	0.156d	0.450e
P1 (DS)	No barrier	0.377c	3.191a	0.174c	0.495d
	Nylon barrier	0.369c	3.131a	0.179c	0.508 cd
	Solid barrier	0.356d	2.953b	0.183bc	0.525bc
P2 (DS)	No barrier	0.467ab	2.739c	0.193ab	0.536b
	Nylon barrier	0.474a	2.678c	0.193ab	0.534b
	Solid barrier	0.460b	2.521d	0.200a	0.557a
P0 (WW)	No barrier	0.160f	2.035f	0.201d	0.548d
	Nylon barrier	0.154f	1.989f	0.213d	0.603c
	Solid barrier	0.149f	1.895f	0.220d	0.614c
P1 (WW)	No barrier	1.040d	3.655bc	0.315c	0.807b
	Nylon barrier	0.968e	3.474de	0.329c	0.838b
	Solid barrier	0.940e	3.398e	0.330c	0.841b
P2 (WW)	No barrier	1.344a	3.943a	0.382b	0.956a
	Nylon barrier	1.258b	3.746b	0.396ab	0.966a
	Solid barrier	1.165c	3.569 cd	0.416a	0.988a
Anova	W	***	***	***	***
	P	***	***	***	***
	C	***	***	***	***
	W*P	***	***	***	***
	W*B	***	ns	ns	ns
	P*B	**	ns	ns	ns
	W*P*B	***	ns	ns	ns

*N* = 5. Values followed by different lowercase letters (a, b) indicate significant difference (*P* < 0.05). W (water treatment), P (phosphorus treatment), C (cropping pattern). ***, **, *, ns, significant difference at 1‰, 1%, 5% and not significant at 5% level

## Discussion

### Plant–plant interaction patterns and their shifts along a gradient of phosphorus and water availability

Plant–plant interactions have long been recognized as crucial drivers of community dynamics and composition, which are highly variable and context dependent [28]. In this study, intercropped species showed a net productivity advantage over monocultures [29, 30]. A biologically diverse community can ameliorate abiotic conditions and facilitate plant growth and development from assembly under stressful conditions [31]. The most important finding of our experiment is that the plant–plant facilitation intensity was stronger under P– and water–deficient conditions and decreased with increasing P and water availability, which indicates that the interspecific interactions were species specific and environmentally dependent [32, 33].

The three performance indicators were used in our study to evaluate the major aspects of plant demographical processes, which can provide a complete assessment of plant interactions. Our findings are consistent with the general observation that maize is the dominant species in intercropping systems, with a higher NE and RII and lower RCI, which indicated the competitive advantage of maize over grass pea in intercropping systems [34, 35]. Interestingly, the yield RII of maize was higher than the biomass RII, while the grass pea yield RII was lower than the biomass RII. This phenomenon was inseparable from the changes in the adaptive strategies of the two species under different environmental conditions [36].

Willey concluded that intercropping increased grain yields over monoculture by 14% and 93% for well-watered and drought stressed conditions, respectively [37]. Similarly, our study confirmed that the positive plant–plant interactions were stronger under drought stress than under well-watered conditions. In practical terms, the facilitative effect was stronger under low-P conditions and the competitive effect was dominant in soils in which sufficient P was present, which indicated that the rhizospheric soil available P level affected the interspecific interaction patterns. For instance, Wang found that interspecific competition intensity was strengthened under high-P conditions and that the RII was higher in P-deficient soils [38]. These findings showed that intercropping systems can achieve the greatest facilitative effect under resource–constrained conditions [39].

In addition, the RCI further explained the interaction variability of both species under different environmental conditions. The interspecific interactions of maize were dominated by intraspecific competition, and the interspecific competition from grass pea was weak, which was attributed to asymmetric interspecific competition [40, 41]. Grass pea was less competitive and received strong interspecific competition from maize, which led to a negative effect on grass yield, whereas the interspecific competition for grass pea biomass was relatively weak. Consequently, for maize, the interspecific competition for soil nutrients was weaker than the intraspecific competition in a monoculture system. Due to the optimized use of resources, yield and biomass were higher in such mixed crops than in monocultures under the same level of nutrient and water inputs, particularly under low-P fertilization conditions. However, the presence of the nylon and solid barriers altered the interspecific interactions. The intraspecific interaction of maize was enhanced due to the restriction of the maize root system activity. At the same time, the barrier decreased the asymmetric competition between the two species and reduced the interspecific competition intensity of grass pea [1, 42].

### Driving mechanism of plant–plant facilitation in response to resource availability

Biologically diverse communities have been shown to utilize P resources more sufficiently than less diverse communities [43]. Interspecific facilitation of P accumulation occurs when one species increases soil P availability and the intercropped companion species can benefit from this process [44, 45]. P0-P1 stimulated the mineralization process of the grass pea rhizosphere, which increased P utilization from mineralized organophosphorus. Therefore, the responses of plant–plant interactions across environmental gradients are variable, and the facilitative interaction by the complementary utilization of P was strongest under P-deficient environments [46]. Conversely, sufficient P availability in P2 suppressed organophosphorus mineralization, and consequently, the interspecific complementary utilization of P was relatively low, which led to a decline in the strength of the plant–plant facilitative effect. Based on these findings, this study highlights an ecological understanding of facilitation under stressful conditions and bridges a gap in interspecific interaction theory in agroecology research [23, 47].

Root isolation indicated that the intensity of rhizosphere interactions played a crucial role in determining plant growth [13, 14]. In this study, maize was competitive due to its larger individual size and strong resource acquisition capabilities, which was in line with the finding that cereals are more competitive than legumes under high levels of resource availability [40, 41]. In particular, grass pea has strong nitrogen fixation and organophosphorus mineralization capability, especially under resource scarce conditions, which could promote the growth of their neighbours [48]. This study elucidated the complementary utilization of water, nutrients, and living space, and the ability of the crop roots to extract nutrients peaked when there was no barrier between both species. Therefore, the maize roots were able to obtain more available resources which resulted in a greater yield and biomass advantage. However, due to strong competition from the maize root system, the grass pea yield and biomass were negatively affected.

The nylon barrier restricted direct rhizospheric interactions between the species, but water, mineral nutrients and root exudates were able to pass through the nylon barrier. Therefore, the nylon barrier limited the extension of the maize root system to the grass pea root system in response to the high resource requirement of maize. Due to the larger individual size of maize, the grass pea plants utilized fewer nutrient resources for plant growth and development, and the excess nutrients and water diffused and travelled through the nylon barrier, which improved the nutrient status of the maize rhizospheric soil. This promotion effect was significantly lower than the direct rhizospheric interactions active under the no–barrier condition. In contrast, grass pea growth was better when it was protected by a nylon barrier in comparison to the no–barrier condition because the nylon barrier weakened direct competition from maize for nutrient acquisition by the grass pea root system. The solid barrier fully restricted rhizospheric interactions between the two plants [18, 49], and the plant roots and soil nutrients could not penetrate the barrier. This diminished the complementary rhizospheric effects. Consequently, the solid barrier significantly decreased the positive effects on maize growth.

Root-induced pH change is one of the major processes that influences P availability in soils [50], and rhizosphere acidification by P-efficient species results in a low pH in the rhizosphere, which increases the availability of insoluble inorganic P in the soil [51]. In this study, variations in rhizospheric pH under different environmental conditions was closely linked to the availability of Olsen-P and interspecific interactions in P-deficient soils. Grass pea acidified its rhizosphere more intensely than maize, and the maize rhizospheric pH was significantly lower when no barrier was present than when a solid barrier was present under P0 conditions. Alternatively, the availability of Olsen-P in the rhizosphere of maize was significantly higher under no–barrier treatment than under the solid barrier treatment, which indicated that the rhizospheric pH of maize was affected by direct interactions with the grass pea rhizosphere and that the variation was restricted by the presence of a solid barrier. However, the enhancement of maize rhizospheric soil P availability was environmentally dependent due to the improvement in P utilization that occurred in low-P soils. These data are in accordance with previous findings that reported a significant increase in rhizospheric P availability of legume and cereal intercrops under P-deficient soil conditions [23].

## Conclusion

Our study revealed that facilitation occurs more frequently under stressful conditions, while competition is stronger under sufficient soil P and water conditions in the maize-grass pea intercropping system. Notably, plant–plant interactions are species specific, and direct rhizospheric interactions play a vital role in determining agroecosystem productivity. The presence of a root barrier elicited the opposite effect on the growth and development of maize and grass pea. Direct root interactions resulted in better plant development and resource acquisition, the nylon barrier partially restricted direct rhizospheric interactions, and the solid barrier fully restricted interspecific rhizospheric interactions. The mechanism by which biodiversity improves plant growth is by strengthening the complementary use of limited nutrients and spatial niche resources. Grass pea was beneficial for maize growth because of its strong ability to mineralize and acidify rhizospheric P under P-deficient conditions. In particular, asymmetric interspecific interactions strengthened maize growth and development. This study highlights the key role of rhizospheric interactions on plant–plant interaction patterns and provides a mechanistic understanding of the facilitative relationship between species diversity and productivity. Further research is needed to more accurately evaluate the ecological theory of species specificity and the tradeoffs between plant–plant interactions in biodiverse systems experiencing ongoing environmental changes.

## Materials and methods

### Experimental statement

A local and widely planted maize variety (*Zea mays* L.) and grass pea (*Lathyrus sativus* L.) were used in this study. The seeds for maize (cv. “Shendan 16”) and grass pea (cv. “Dingxi”) were obtained from Lanzhou University. All plants were grown in pots, and the collection of plants and soil materials were maintained in accordance with the guidelines and legislations of Lanzhou University. The experimental rainout shelter was housed at Lanzhou University. Permission for this research project was obtained from the State Key Laboratory of Grassland Agro-Ecosystems, College of Ecology, Lanzhou University, and this research did not require any other special permits.

### Growth conditions and materials

Experiments were conducted at the Yuzhong Experiment Station of Lanzhou University in Yuzhong County, Gansu Province, China (35°51′N, 104°07′E, altitude of 1620 m) during the growing season (March–October) of 2017. The pH, organic carbon content, total nitrogen content, total phosphorus content, available potassium content, available phosphorus content, % CaCO_3_, and EC of the soil used in pots were 8.5, 9.54 g kg^−1^, 0.48 g kg^−1^, 0.62 g kg^−1^, 82 mg kg^−1^, 6.8 mg kg^−1^, 8.5%, and 0.45 mS cm^−1^, respectively. The pots were kept under an open rainout shelter (50 m long × 24 m wide × 5.7 m high), the shelter was covered during precipitation events.

Seeds were sterilized in 5% sodium hypochlorite for 30 s, washed several times with sterile water, and sown directly into the soil. One maize and five grass pea seeds were planted in the intercropping pots, and two maize and ten grass pea seeds were sown in the monoculture pots. Each plastic pot, which had dimensions of 0.3 m diameter × 0.4 m height and did not contain drainage hole, was filled with a 14 kg mixture of silty-loam loess soil (ustorthents according to U.S. soil taxonomy) and vermiculite (soil: vermiculite = 2: 1, v/v) and had a pot capacity (PC) of 32.5% [52]. Before planting, a nutrient solution containing 10 g of NH_4_NO_3_ and the corresponding P gradient was applied to each pot.

### Experimental design

**Fig. 9:**
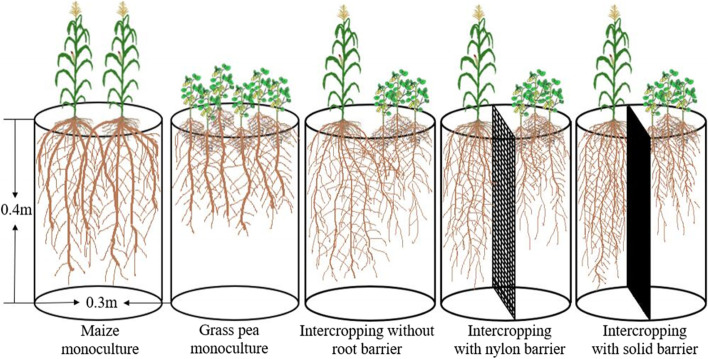
Schematic diagram illustrating five different cropping patterns

The experiment followed a three-factor design with five replicates. The main plot included five cropping patterns, including maize and grass pea grown in separate pots as monocultures, maize and grass pea grown in the same pot as intercrops without a root barrier, maize and grass pea intercrops with a nylon net barrier (30 μm), which prevented interspecific root intermingling but permitted the exchange of root exudates and solutes, and maize and grass pea intercrops with a solid barrier to completely eliminate root contact and solute movement (Fig. 9). The three P application rates were 1) P0 (without P addition, CK), 2) P1 (1.5 g P as phytate (C_6_H_18_O_24_P_6_) pot^−1^),), containing an organic insoluble form of P, and 3) P2 (1.5 g P as KH_2_PO_4_ pot^−1^), containing a highly soluble form of P. The two water treatments were: 1) drought stress, 40% PC (DS), and 2) well-watered, 80% PC (WW). In this study, before imposing drought–stressed conditions, all pots were kept well–watered (80% PC). The water treatments were initiated at the maize trefoil stage, and the soil water content was controlled gravimetrically by weighing and applying water to the top of the pots daily during the late afternoon throughout the growing period until maturity.

### Plant and soil sampling and chemical analysis

#### Plant sampling

At maturity, maize and grass pea plants were collected (grass pea reached a mature stage in mid-July, and maize reached a mature stage in the beginning of October). The plants were carefully uprooted and divided into three parts: belowground portions (root biomass), shoot portions (leaf and stem biomass) and grain (seed biomass). These samples were oven-dried at 80 ℃ until they reached a constant weight.

### Measurement of rhizospheric soil total phosphorus (STP), Olsen-phosphorus (Olsen-P), and pH

At maturity, the rhizospheric soils of maize and grass pea were collected by brushing off plant roots and passing the soils through a 2-mm mesh sieve. Half of the soil was air-dried to determine the STP, Olsen-P and pH, and the remainder was immediately stored at 4 °C for the determination of phosphatase activities. STP was determined colorimetrically after digestion with perchloric acid. Olsen-P was determined using the Olsen method [53]. Soil pH was measured in a 1:2.5 soil: water suspension. Phosphatase activities were determined according to a method by Tabatabai and Bremner and expressed as mg PNP kg^−1^ soil h^−1^ [54]. All calculations were expressed on an air-dried basis.

### Water use efficiency (WUE)

WUE was calculated as the ratio of total yield and biomass of the two species to the sum of total water consumption during the growing season and the difference in the soil water content of the pot profile between the beginning and end of the growing season.

### Calculation of response variables in the intercropping system

The net effect (NE) is calculated as the difference between the observed yield and the expected yield using following equations:

1 $$NE=p_1NE_1+p_2NE_2=(Y_1-p_1M_1)+(Y_2-p_2M_2)$$

where Y_1_ and Y_2_ are the observed yields and biomasses of maize and grass pea in the intercropping system, M_1_ and M_2_ are the yields and biomasses of maize and grass pea in the monocultures, and p_1_ and p_2_ are the land use proportions of the two species during intercropping, where p_1_ + p_2_ = 1 [29].

The relative interaction index (RII) is used to evaluate the nature and strength of plant–plant interactions. It can be quantified as follows:

2 $$RII=(Y_1-p_1M_1)/(Y_1+p_1M_1)+(Y_2-p_2M_2)/(Y_2+p_2M_2)$$

When RII for a species is > 0, the net interaction is facilitative; when it is equal to 0, there is a neutral interaction; and when it is < 0, the net interaction is competitive [25].

Relative competition intensity (RCI) indicates the competitive ability of a species. It can be calculated according to grain yield or biomass production under a given crop combination. In the present study, the RCI was calculated using the following equations:

3 $$RCI=(p_1M_1-Y_1)/(p_1M_1)+(p_2M_2-Y_2)/(p_2M_2)$$

A value of RCI = 0 indicates that interspecific competition equals intraspecific competition. A value of RCI > 0, indicates that interspecific competition is higher and intraspecific competition is lower, and vice versa [55].

### Data analyses

The mean values of the treatments were compared using the least significant difference at p < 0.05. One-way ANOVA was used to analyse the effects of cropping patterns, phosphorus levels, and water availability on NE, RII, RCI, phosphatase activity, rhizospheric soil phosphorus, pH, yield, biomass, N uptake, P uptake, and water use efficiency. Three-way ANOVA was used to test the interactions between planting patterns, phosphorus levels, and water gradients. The data were analysed using the SPSS (IBM SPSS 26.0 version, Chicago, IL, USA) software. The figures were plotted using Origin v. 9.0 software.

## Supplementary Information

**Additional file 1**: **Figure S1.** Schematic diagram illustrating five different cropping patterns. **Figure S2. **Biomass allocation of maize and grass pea in response to three P treatments (P0, Phytate and KH_2_PO_4_) and two water treatments (drought stress (DS) and well-watered (WW)) under three root barrier conditions (no barrier, nylon barrier and solid barrier) in maize-grass pea intercropping system

## Data Availability

The datasets generated and analysed during the current study are not publicly available because the data are still being supplemented and improved to be published as part of a graduation thesis at later stage, but they are available from the corresponding author upon reasonable request.
