# Supplementary material for: Transition in plant–plant facilitation in response to soil water and phosphorus availability in a legume-cereal intercropping system
Source: BMC Plant Biol. 2022 Jun 28;22:311. doi: 10.1186/s12870-022-03706-6 (PMC9238078; doi:10.1186/s12870-022-03706-6)
Supplement: Supplementary file 1 — Additional file 1: Figure S1. Schematic diagram illustrating five different cropping patterns. Figure S2. Biomass allocation of maize and grass pea in response to three P treatments (P0, Phytate and KH2PO4) and two water treatments (drought stress (DS) and well-watered (WW)) under three root barrier conditions (no barrier, nylon barrier and solid barrier) in maize-grass pea intercropping system [file 12870_2022_3706_MOESM1_ESM.docx]

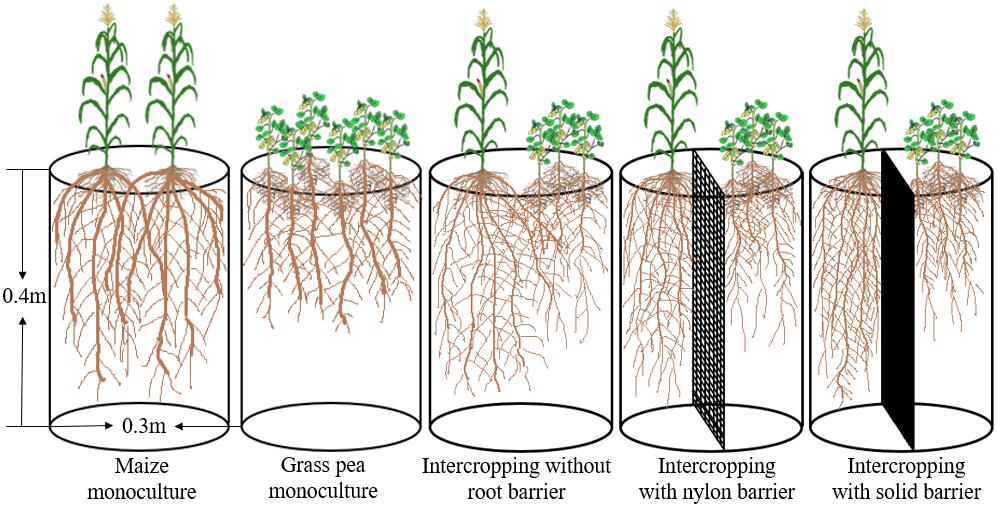


Fig. S1 Schematic diagram illustrating five different cropping patterns.



Fig. S2 Biomass allocation of maize and grass pea in response to three P treatments (P0, Phytate and KH_2_PO_4_) and two water treatments (drought stress (DS) and well-watered (WW)) under three root barrier conditions (no barrier, nylon barrier and solid barrier) in maize-grass pea intercropping system.
